# Digital Detox Among Adolescents at Summer Camp: Nursing Implications

**DOI:** 10.1097/CNJ.0000000000001280

**Published:** 2025-12-08

**Authors:** Janet M. Reed, Ashley Gies

**Affiliations:** **Janet M. Reed, PhD, RN, CMSRN,** is an assistant professor of nursing at Kent State University. Her research interests include the social, emotional, and pedagogical effects of technology. She enjoys serving as a camp nurse.; **Ashley Gies, BSN, RN, CPN, CPHON,** is a pediatric nurse working at Akron Children's Hospital who is completing a master's degree at Kent State University.

**Keywords:** adolescents, camp, camp nursing, nursing, pediatric nursing, spiritual care, technology addiction, technology overuse

## Abstract

Computer and internet addiction as well as compulsive social media use have become significant problems for adolescents. Residential summer camps offer the opportunity for campers to experience a *digital detox* in a supportive setting where digital devices are not available. A retrospective chart analysis of health clinic logs for campers ages 8-18 at an 8-day residential Christian athletic summer camp in 2023 asked: What percentage of summer camp health clinic visits were due to symptoms consistent with technology withdrawal, and did health clinic utilization differ based on gender? Visits for sleep (6%) and anxiety (2%) symptoms had the highest likelihood of being consistent with symptoms of technology withdrawal; female campers had more clinic visits than males. Nursing interventions for camp health concerns and the relevance of biblical spiritual care for adolescent campers are discussed.

**Figure:**
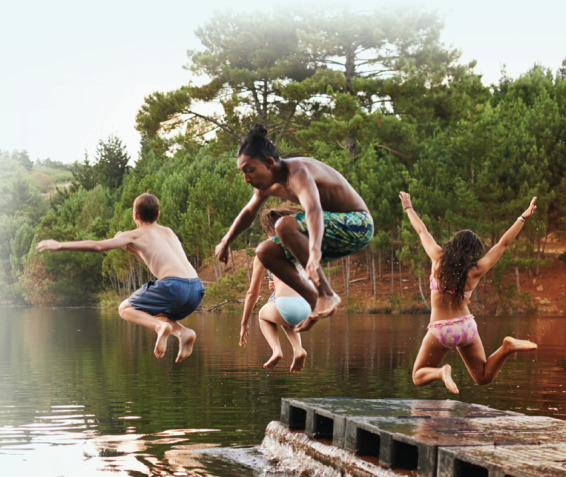
No caption available.

The last decade has seen a rise in both mental health concerns in children and adolescents, and their use of digital technologies (Haidt, 2024; Odgers & Jensen, 2020). Are these two occurrences linked? Technology, often used positively for entertainment, education, and social messaging, can lead to negative consequences when used in excess and has been linked to digital addiction in children (Çimke et al., 2023). Concerns about technology overuse are frequent among parents, educators, and communities as a variety of health consequences can ensue. Discussions about the adverse effects of smartphones, gaming, and social media are occurring in tandem with escalating concerns regarding the potential worsening mental health of adolescents.

According to the American Camp Association (n.d.), more than 26 million children and adults participate in camp experiences each summer. In an ever-increasingly digital world, youth attending residential summer camp may experience a sudden detachment from these technologies, termed a *digital detox*, which may lead to physical and mental health symptoms. A digital detox is an intentional unplugging or disconnection from one or all electronic devices, similar to fasting (Radtke et al., 2022). Residential or overnight summer camps offer a safe, supportive setting where children and adolescents can experience a natural digital detox from the usual technologies in their lives. Camp nurses, staff, and counselors work to support campers' physical, mental, emotional, social, and spiritual development during a digital detox. The purpose of this research was to examine the most common physical and mental health symptoms experienced by campers at a large Christian residential summer camp while undergoing a digital detox by using a retrospective analysis of de-identified health clinic logs.

## TECHNOLOGY USE AND OVERUSE

During the COVID-19 pandemic, screen time among children and teens increased by 52% (Madigan et al., 2022). The Centre for Addiction and Mental Health (CAMH, 2018) surveyed adolescents in the province of Ontario, Canada, and learned that 94% of students visit social media sites daily, with 23% reporting 5 or more hours of social media each day.

Technology addiction can be defined as “compulsive use that is not necessary, accompanied by some impairment in health or social functioning” (Clements, 2021, p. 195). Internet addiction has been associated with mental health conditions including loneliness, poor self-esteem, anxiety, depression, and lack of sleep (Kawabe et al., 2016). A related concept is technology *overuse*, the excessive use of technology that is not necessarily compulsive or addictive but is similarly characterized by high usage time that can negatively affect various aspects of life (Serenko & Turel, 2022). Overuse of digital devices such as smartphones, computers, and video games has been significantly associated with internet addiction which can negatively impact mood, relationships, academic performance, sleep, and life satisfaction (Çimke et al., 2023). Panova and Carbonell (2018) reviewed studies on smartphone behaviors and concluded that research findings do not meet the criteria for addiction, but overuse can be considered “problematic or maladaptive use” (p. 253).

Nicholas Carr's 2011 book, *The Shallows: What the Internet Is Doing to Our Brains*, explored how the internet and digital technology are reshaping humans' brains and cognitive processes. With constant distractions and bite-sized bits of information, Carr argues that the internet is altering society's ability to concentrate deeply, think critically, and reflect contemplatively by reshaping the neural networks in our brains. Another societal concern is that increasing use of technology and social media can cause people to lose opportunities for face-to-face interaction. In *Alone Together: Why We Expect More from Technology and Less from Each Other*, Turkle (2017) discusses technology's impact on human relationships, examining the increasing reliance on digital devices and social media that is producing the paradox of being *alone together*, that is, feeling both connected and disconnected in an increasingly digital world. High levels of loneliness have been reported globally with 9.2% of adolescents (ages 12-17) reporting loneliness (Surkalim et al., 2022).

Technology overuse can result in mental, behavioral, physical, and social consequences. Meta-analyses have documented the impact of screen time on adolescents' mental health, causing anxiety, depression, and behavioral problems such as aggression and attention-deficit/hyperactivity disorder symptoms (Eirich et al., 2022; Madigan et al., 2022). Khalaf et al.'s (2023) systematic review of data on smartphone and social media use among teenagers revealed increased mental distress, self-harming, and suicide tendencies. Funk et al. (2023) reported that psychotropic medications (i.e., antidepressants, ADHD, bipolar, and anxiety medications) are the second most common classification of camper medications after allergy/antihistamine medications. Some campers may be taken off ADHD medications during the summer; this can contribute to an increase in problematic behaviors (Schmidt, 2022).

Technology overuse also can contribute to physical problems. The higher the screen use time by adolescents, the lower their physical activity and greater their obesity rates (Nagata et al., 2023). In a large study of adolescents in Canada, only 23% of students between grades 7 and 12 met the recommendation for daily physical activity (CAMH, 2018). Insufficient sleep (Twenge et al., 2017), unhealthy dietary choices (Ryu et al., 2022), and dysfunctional eating patterns among adolescents (Fiedler et al., 2023) are documented outcomes from two or more hours of daily screen time. Studies confirm that screen time increases with age throughout adolescence; results are inconclusive about gender differences in internet addiction (Kawabe et al., 2016; Madigan et al., 2022).

Though using technology to stay socially connected also produces positive outcomes in health and well-being (Vuorre & Przybylski, 2024), technology overuse among children and adolescents can result in their withdrawal and disconnection from nature (Yao & Liu, 2022). Michaelson et al. (2020) reported how technology overuse correlates with a diminished sense of the importance of nature, including the addictive characteristics inherent in technology and the prevailing adolescent notion that indoor settings are more comfortable and safe. However, upon disengaging from devices, adolescents often regain appreciation for the outdoors (Michaelson et al., 2020). This is where camps benefit adolescents.

## CAMPS AND DIGITAL DETOX

**Figure 1.**
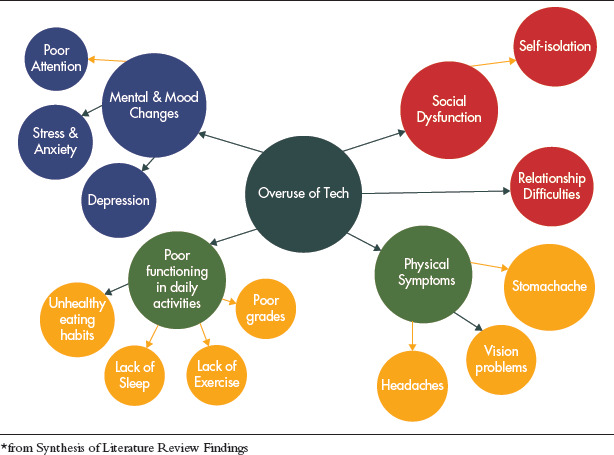
Potential Outcomes of Technology Overuse∗

The potentially negative impacts of technology overuse in children and adolescents are well documented in the literature. Figure 1 summarizes these potential issues linked to technology overuse from a synthesis of literature. Campers naturally experience a digital detox at overnight summer camp, which can produce technology withdrawal symptoms such as anxiety and disturbed sleep. Overnight camps offer campers a connection with nature as well as a digital detox. A digital detox refers to a period during which an individual ceases to use electronic devices: smartphones, computers, and tablets. The goal of a digital detox is to disconnect from the constant barrage of notifications, information overload, and screen time that is prevalent in modern life. During a digital detox, people often engage in activities that promote mindfulness, relaxation, and offline social interactions which might include spending time in nature, engaging in hobbies, and having face-to-face social conversations. Unplugging from technology can reduce stress, improve focus and concentration, deepen sleep, and offer a greater sense of presence and connection with one's surroundings. A digital detox can specifically benefit adolescents by stimulating increased creativity, enhancing social skills, offering more physical activity, and improving mental well-being (Gaurav, 2024).

Despite the positive long-term benefits of a digital detox, in the short-term, temporary emotional and physical symptoms of technology withdrawal can emerge. Because a behavioral addiction involves engaging in a specific behavior for relief, comfort, or stimulation, discontinuing that behavior may result in discomfort or unease. Compulsive behaviors can stimulate the release of dopamine, a neurotransmitter associated with pleasure and reward (Substance Abuse and Mental Health Services Administration [SAMHSA], 2012). Consequently, prolonged exposure to dopamine alters the functioning of various brain regions, including the amygdala, amplifying negative emotions such as fear, anxiety, and stress once the compulsive behavior ceases (SAMHSA, 2012). This leaves addicted individuals feeling compelled to resume the behavior to alleviate the discomfort of withdrawal. Clements (2021) describes a tool used to assess the negative emotions experienced during technology withdrawal such as feeling annoyed, being afraid of missing out, and experiencing anxiety as well as mental distress. Stanovsek (2018) confirmed that when smartphones aren't being used to ease social stress, feelings of uneasiness, boredom, and anxiety may arise. These feelings may be the strongest during the first few days away from technology (Coyne & Woodruff, 2023).

Summer camps offer adolescents opportunities to reconnect with the natural world and foster creativity, reduce stress, and promote healthy development. Louv (2008) notes that, “in nature, a child finds freedom, fantasy, and privilege; a place distinct from the adult world, a separate place” (p. 7). Though campers may initially crave their devices or experience technology withdrawal, Kronk (2019) noted that eventually they feel relieved and positive after a camp digital detox. Megret (2023) found teenagers were overwhelmingly positive about a tech-free camp experience, especially regarding face-to-face social interactions. In an increasingly digitalized world, few places other than camps allow adolescents the chance to disconnect from technology and reconnect with nature and other people in a supportive environment. Promoting enjoyment of nature aligns with God's acknowledging that all he made is very good (as discussed in Genesis 1).

Warner and Wycoff (2023) surveyed summer camp staff about the most anticipated challenges for campers and found that the top five themes related to camper behavior were behavior management, difficult behavior, mental health challenges, developmental delays, and social/emotional issues such as anxiety. Other researchers have examined injury patterns and mental health among campers; however, a search of current literature revealed no articles specifically analyzing symptoms reported by campers related to technology withdrawal. Given concerns about increasing technology use and potential problems with technology withdrawal, this study sought to provide information on the experience of digital detox in adolescents attending a summer residential camp.

## STUDY METHODS

The two research questions guiding this study were 1) What percentage of summer camp health clinic visits were due to symptoms consistent with technology withdrawal? and, 2) Did health clinic utilization differ based on the gender of the camper?

After Institutional Review Board (IRB) approval, the authors partnered with a large Christian athletic camp to perform a retrospective chart analysis of de-identified health clinic logs for an 8-day term in the summer of 2023. Other research projects have similarly used completely de-identified health data for camp research (Kolberg et al., 2020). De-identified health data were coded for analysis by the study authors and entered into the Statistics Package for the Social Sciences (SPSS) program for data analysis. Researchers classified symptoms that brought campers to the health clinic along with the date, time, cabin number, and camper's gender. Descriptive and statistical data analysis were performed in SPSS to answer the research questions.

## DATA ANALYSIS

In total, 441 visits were logged in the clinic and were de-identified and coded. The sample included camp health data from a total of 307 campers ages 8-18. The sample included boys (*n* = 153) and girls (*n* = 154) attending a Christian sports camp for 8 days. Of the total campers, 166 (54%) visited the health clinic at least once. Among female campers, 31.8% took routine medications compared to 25.5% of boys. Routine medication visits were not included in analysis; only clinic visits for a specific health complaint were analyzed.

The camp health clinic had a mean of 49 visits per day with a maximum of 84 visits on the seventh day. Nineteen percent of campers (19%, *n* = 58) were considered “frequent flyers” who visited the clinic three or more times during the 8 days. Visits to the clinic by frequent flyer campers made up 35% of all health clinic visits. Some campers returned for the same need multiple times, such as wound bandages needing to be changed, or musculoskeletal injuries needing pain management.

**Figure 2.**
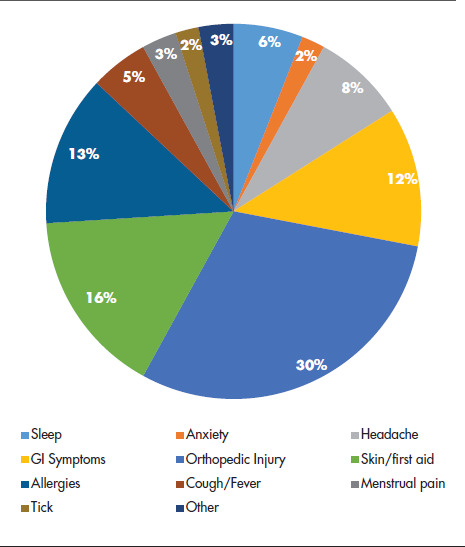
Symptoms Bringing Campers to Health Clinic by Percentage of Incidence

The first research question examined what percentage of health clinic visits were due to symptoms consistent with technology withdrawal. The percentages of symptoms that brought campers to the health clinic were as follows: orthopedic injuries (30%); skin/first aid (16%); allergies (13%); GI symptoms (12%); headache (8%); sleep (6%); cough/fever (5%); menstrual pain (3%); other (3%); anxiety (2%); ticks (2%; see Figure 2). Based on literature related to digital detox symptoms, sleep and anxiety had the highest likelihood of being consistent with symptoms of technology withdrawal; these two symptoms combined comprised 8% of clinic visits. Furthermore, older adolescent campers had higher rates of clinic visits for sleep problems than younger campers. As researchers have found that screen time increases with age throughout adolescence (Kawabe et al., 2016; Madigan et al., 2022), it is of interest that the older teens had higher rates of clinic visits related to sleep concerns—a symptom that could be related to technology withdrawal.

Camper visits for sleep and anxiety symptoms peaked on the third and fourth days of camp. Similarly, the first few days during a digital detox are when most research studies provide interventions due to emergence of withdrawal symptoms (Radtke et al., 2022). The classifications of headache and gastrointestinal symptoms also had possible connections to technology withdrawal, although these could be related to other situations such as dehydration, tiredness, missing home, or constipation. All other problems leading to health clinic visits were deemed to have an unlikely association with technology withdrawal.

The second research question addressed gender differences among campers using the health clinic. More female campers (*n* = 94) utilized the health clinic than male campers (*n* = 72) during the 8 days. An independent samples *t*-test was conducted to compare the mean number of visits between males and females. The results revealed a statistically significant difference: (*t*(163) = 2.36, *p* = .02). Female campers (M = 3.0, SD = 2.6) had a significantly higher mean number of visits during the camp compared to male campers (M = 2.1, SD = 1.8).

It is possible that gender may influence the healthcare-seeking behavior of campers, with females exhibiting a higher utilization of health clinic services. Suoniemi et al. (2021) reported that girls were more likely to report their symptoms and seek help when using school nurse services. Females may be more inclined to discuss emotional concerns with others, whereas some boys may perceive seeking healthcare as a sign of weakness or may fear the stigma associated with certain health issues.

## DISCUSSION

Residential camps have reported increased mental and behavioral challenges among campers which led this research team to examine the most prevalent symptoms bringing campers into the health clinic. Unsurprisingly, the top complaint at this athletic camp was orthopedic injuries (30% of visits). This is consistent with a large study by Handler et al. (2018) on camp injury and illness which reported sports as the most frequent cause of injury at summer camp. Sleep and anxiety complaints, the symptoms most associated with possible technology withdrawal, accounted for 8% of total visits.

Campers often have difficulty sleeping at residential summer camps. Six percent (6%) of clinic visits were related to sleeping difficulty. Camp nurses can help promote proper sleep hygiene practices for campers and teach campers and counselors nonpharmacological methods to improve their sleep such as relaxation techniques; avoiding caffeine, chocolate, and sugary foods in the afternoon and evening; and providing ear plugs, eye masks, or white noise machines for campers who are environmentally sensitive.

Anxiety complaints at the health clinic were low (2%) but were likely underreported as only campers with more severe anxiety (e.g., panic attacks) who could not be handled outside the clinic by counselors were captured in the data. More campers may have experienced lower to moderate levels of anxiety. Camp nurses have a pivotal role in educating counselors on how to handle and respond to campers with anxiety symptoms (e.g., teaching deep breathing and relaxation methods). For campers experiencing panic with hyperventilation, nurses should teach and demonstrate pursed-lip or diaphragmatic breathing techniques, as well as intermittent breathing into a paper bag to increase carbon dioxide and reduce symptoms. Nursing intervention for campers with anxiety includes mindfulness training and distraction which may help increase self-regulation and reduce emotional distress behaviors at camp (Opalinski & Martinez, 2021). Additionally, objects such as stress balls or calm-down jars (filled with water, glitter, clear glue, and food coloring that students shake and then focus on) can be helpful to campers coping with anxiety (Opalinski & Martinez, 2021).

Physical complaints such as headaches, nausea, stomachaches, and insomnia have been linked to technology withdrawal (Eide et al., 2018). In our study, headache complaints made up 8% of clinic visits. At camp, headaches also could be due to dehydration. A camper with a headache should be assessed for possible concussion which commonly occurs during sports such as soccer, basketball, and swimming (Kolberg et al., 2020). Gastrointestinal symptoms such as vague abdominal pain or nausea may be related to dietary changes at camp, stress/anxiety, or missing home which often presents this way in younger campers (Demetriou et al., 2022). Missing home can result in various somatic complaints and has been associated with depression and anxiety in children (Demetriou et al., 2022). Nursing interventions for campers who are missing home include offering empathetic listening, validation, encouragement, assistance with socialization, and distraction techniques.

The symptoms experienced during a digital detox vary depending on individual factors, such as the extent of an adolescent's digital dependence and personal resilience. Personal resilience among adolescents refers to their succcessful adaptation to adversity, challenges, or stressful situations (Mesman et al., 2021). Resilience determines teens' capacity to bounce back from setbacks, maintain a positive outlook, cope effectively with difficulties, and develop skills to navigate social and emotional challenges. An empowering relationship with an adult, such as a camp nurse or counselor, can positively impact campers' resilience (Ungar, 2013).

In addition to resilience, a relationship with a camp nurse can foster campers' spiritual development. Shim (2019) noted that in previous research on adolescents and smartphone addiction that “understanding the biblical image of God and spiritual well-being closely relate to preventing addiction” (p. 1281). Christian nurses who cultivate spiritual well-being and resources in campers can strengthen campers' boundaries for appropriate use of technology. Shim's study of smartphone user addiction among youth showed that adolescents with higher risk for smartphone addiction had lower levels of spiritual well-being, highlighting the potential for spiritual interventions. Nursing interventions to support spiritual development of campers undergoing digital detox might include corporate opportunities of praise, worship, and prayer, as well as offering compassionate care, guiding mindfulness activities, creating safe spaces for quiet reflection, sharing inspirational stories and Scripture, and promoting an appreciation of nature as God's creation.

### Study Limitations

It should be noted that camps are diverse and this research was performed at only one Christian sports camp, so the results are not generalizable to all camps. This athletic camp offers daily sports competitions, outdoor adventure trips, and water activities, so we anticipated more physical injuries and orthopedic problems than a camp without an athletic focus. Also, we examined only an 8-day term, whereas other camp lengths may have variations in the data.

We did not capture subclinical concerns that campers might have been experienced with counselors outside the health clinic. We also did not collect data on campers' technology usage before coming to camp. The study did not measure or quantify all the psychiatric symptoms of campers, such as irritability, restlessness, boredom, or social withdrawal, which counselors might observe. Instead, we only quantified symptoms such as headaches, sleeplessness, anxiety, etc. that led to visits to the health clinic. Therefore, the true prevalence of tech withdrawal is unknown and likely underrepresented in this study. Symptoms of withdrawal from smartphones have not been well-studied in the literature (Aarestad et al., 2023). Smartphones are now so widespread that conducting true experimental research with random assignment to different technology exposure groups is nearly impossible. As a result, much of the reported research relies on quasi-experimental and correlational studies, limiting the ability to draw strong causal inferences without longitudinal studies (Dienlin & Johannes, 2020).

## NURSING IMPLICATIONS WITH BIBLICAL PERSPECTIVE

The American Academy of Pediatrics (2023) no longer places a set limit for adolescents on media but encourages digital citizenship with parental oversight. Nurses should encourage parents to monitor and moderate daily technology use and provide device-free time and media-free zones (e.g., bedrooms) along with sufficient physical activity. The website HealthyChildren.org offers a customizable family media plan to help parents and children set appropriate boundaries on technology usage. Conflicting information in the literature about the amount of daily smartphone use is problematic. Although some researchers suggest negative health outcomes increase with 2 hours of daily screen time (Ryu et al., 2022; Twenge et al., 2017), other researchers have reported that adolescents using smartphones 2-4 hours/day showed no increased adverse health outcomes compared to non-users (Cha et al., 2023).

The Bible gives wisdom about the value of moderation, such as 1 Corinthians 6:12 (ESV): “All things are lawful for me, but not all things are helpful.” Though technology offers many positive aspects, its use must be moderated with self-control and discernment. Consider 1 Thessalonians 5:21-22 (ESV): “But examine everything; hold firmly to that which is good; abstain from every form of evil.” Though technology itself is not evil, anything that controls us or separates us from God's best for us can be detrimental. Parents, nurses, and camp leaders can model prudent use of technology while promoting healthy coping strategies, face-to-face relationships, and outdoor experiences. Christian parents and camp nurses can help adolescents consider how they might turn to God rather than technology when seeking answers, coping, or stress relief. Wisdom is not in technology, but in Christ “in whom are hidden all the treasures of wisdom and knowledge” (Colossians 2:3, ESV).

Knowing that summer camp can be a hectic and exhausting environment, camp nurses should emphasize self-care for themselves, camp staff, and the campers they serve (Schmidt, 2022). Nurses should use open, nonjudgmental, and developmentally appropriate tactics with campers who are struggling with various symptoms of digital detox. Additionally, camp nurses can support campers by creating welcoming and inclusive environments, encouraging socialization, and allowing for breaks or alternative activities when campers are feeling highly anxious or overwhelmed. Because boys may not seek healthcare as frequently as girls, camp nurses can encourage boys to return to the clinic for follow-up and should not hesitate to seek campers out to evaluate health concerns and coping effectiveness. Nurses also can collaborate with counselors to engage boys one-to-one if health issues or technology withdrawal behavior are concerning. Galatians 6:2 (NIV) states, “Carry each other's burdens, and in this way you will fulfill the law of Christ.” Camp nurses have a privilege and rewarding opportunity to honor Christ by caring holistically for the campers they serve.

## CONCLUSION

Few places in society offer children and adolescents a true digital detox. Residential summer camps provide one of these rare opportunities, although the experience can lead to temporary technology withdrawal symptoms such as anxiety, sleep disturbances, and somatic complaints. These symptoms, however, have not been thoroughly studied, highlighting a need for future research. Despite this, summer camps play a crucial role in adolescents' positive development by offering a safe environment where they can connect with nature, forge new relationships with peers and mentors, and engage in enriching experiences. Camp nurses hold a unique and vital position in this setting, as they can actively promote the physical, social, psychological, and spiritual well-being of campers. By offering compassionate care and teaching essential life skills for self-regulation, camp nurses help children and adolescents navigate the challenges of digital detox and thrive in their camp experience.

**Acknowledgment:** The authors would like to acknowledge Lynn Holiday, MD, and Ryan McKinney for their assistance with collecting and managing study data.

## Web Resources

- **Alliance for Camp Health**
https://allianceforcamphealth.org/
- **American Camp Association: Pulling the Plug**
https://www.acacamps.org/article/camping-magazine/pulling-plug-why-teaching-campers-how-detach-their-devices-critical-summer-success
- **American Psychological Association: Health Advisory on Social Media Use**
https://www.apa.org/topics/social-media-internet
- **HealthyChildren.org: American Academy of Pediatrics**
https://www.healthychildren.org/English/fmp/Pages/MediaPlan.aspx
- **U.S. Department of Health and Human Services: Youth Mental Health**
https://www.hhs.gov/surgeongeneral/reports-and-publications/youth-mental-health/index.html
